# Valence Variability Induced in SrMoO₃ Perovskite by Mn Doping: Evaluation of a New Family of Anodes for Solid-Oxide Fuel Cells

**DOI:** 10.3390/ma18030542

**Published:** 2025-01-24

**Authors:** Lucía Sánchez de Bustamante, Romualdo Santos Silva, José Luis Martínez, María Teresa Fernández-Díaz, Ainara Aguadero, José Antonio Alonso

**Affiliations:** 1 Instituto de Ciencia de Materiales de Madrid, Consejo Superior de Investigaciones Científicas, Cantoblanco, E-28049 Madrid, Spain; lucia.sb@csic.es (L.S.d.B.); martinez@icmm.csic.es (J.L.M.); ainara.aguadero@csic.es (A.A.); 2 Departamento de Física de Materiales e Instituto Pluridisciplinar, Universidad Complutense de Madrid, E-28040 Madrid, Spain; romualdo.silva@csic.es; 3 Institut Laue Langevin, BP 156X, F-38042 Grenoble, France; ferndiaz@ill.eu

**Keywords:** anode, IT-SOFC, MIEC behavior, NPD, oxygen vacancy, perovskite

## Abstract

We report on a series of SrMo_1−x_Mn_x_O_3−δ_ perovskite oxides designed as potential anode materials for solid oxide fuel cells (SOFCs). These materials were synthesized using a citrate method, yielding scheelite-type precursors with nominal SrMo_1−x_Mn_x_O_4_ compositions, which were further reduced to obtain the active perovskite oxides. Their structural evolution was examined through X-ray diffraction (XRD) and neutron powder diffraction (NPD). These techniques provided insights into the crystallographic changes upon Mn doping, revealing key factors influencing ionic conductivity. Whereas the oxidized scheelite precursors are tetragonal, space group *I4_1_/a*, the reduced perovskite specimens are cubic, space group *Pm-3m*, and show the conspicuous absence of oxygen vacancies, even at the highest temperature of 800 °C. The transport properties were analyzed through electrical conductivity measurements, exhibiting a metallic-like behavior. Thermogravimetric analysis (TGA) and dilatometry give insights into the thermal stability and expansion behavior, essential for SOFC operation. Test single SOFCs were built in an electrolyte-supported configuration, on LSGM pellets of 300 μm thickness, assessing the performance of the title materials as anodes. This work emphasizes the critical relationship between the crystal structure and its electrochemical behavior, providing a deeper understanding of how doping strategies can optimize fuel cell performance.

## 1. Introduction

Over the past few decades, rapid technological and industrial advancements have driven global energy consumption to unprecedented levels. This surge in demand has fueled the urgent search for clean, secure, affordable, and sustainable energy resources. Scientists from various disciplines are collaborating worldwide to address this critical challenge. Traditionally, fossil fuels have been the dominant energy source to meet global needs [1,2]. Unfortunately, the environmental consequences of fossil fuel use, particularly their contribution to climate change, have highlighted the need to explore alternative energy solutions [3,4,5]. Researchers have focused on developing energy resources that are not only clean, efficient, and sustainable but also economically viable and secure, including hydrogen as an energy vector [6,7,8]. Among the promising alternatives, fuel cells meet these criteria and have been commercially available since the mid-1960s.

Fuel cells are static energy conversion devices that generate electricity through an electrochemical reaction between a fuel and an oxidizing agent [9,10,11]. Unlike combustion engines, fuel cells directly convert chemical energy into electrical energy, eliminating the need for a combustion process. While fuel cells are highly efficient, they still produce waste heat and exhaust gases as byproducts of redox reactions. However, through cogeneration, this waste heat can be utilized to generate additional electricity, further improving overall efficiency [12]. Among them, intermediate temperature solid oxide fuel cells (IT-SOFCs) are advanced energy conversion devices capable of achieving efficiencies greater than 70% with cogeneration, making them promising candidates for clean energy applications [13,14]. It is therefore urgent to find ways to optimize their performance in order to improve their overall efficiency. The critical components of SOFCs include the anode, electrolyte, and cathode, while interconnects and sealants become equally significant when constructing SOFC stacks. In SOFCs, all the components are oxides, and the performance of such fuel cells relies on the ionic conductivity of oxide ions across the electrolyte, which requires elevated temperatures, in the 800–1000 °C range [15,16,17,18,19].

Several key challenges impacting SOFC operation are the following [20]: (i) Anode challenges: performance is hindered by sulfur poisoning, coke formation, adsorbate surface diffusion, and charge transfer limitations at the triple-phase boundary (TPB). (ii) Electrolyte issues: Efficient oxygen ion (O_2_^−^) migration is critical for energy conversion but can be restricted by factors such as ion channel design, dopant-induced vacancy behavior, lattice strain, and phase distribution. Optimizing grain and grain boundary conductivity is essential for developing effective electrolytes. (iii) Cathode limitations: Cathodes face multiple rate-limiting factors, including active surface geometry and overpotential. Comprehensive electrochemical impedance spectroscopy (EIS) is required to evaluate and improve cathode performance. (iv) Interconnect constraints: Interconnects must maintain chemical stability in oxidizing and reducing environments while matching the coefficient of thermal expansion (CTE) of surrounding components. These properties are critical for withstanding repeated thermal cycling during SOFC operation. (v) Sealant requirements: Sealants must effectively isolate fuel and oxidizing gases while maintaining CTE compatibility with neighboring components. Additionally, the glass transition temperature of the sealant dictates the upper operating limit of the SOFC system.

Traditional anode materials are constituted by a biphasic product consisting of a mixture of the electrolyte (ceramic) and Ni metal; this material is denominated cermet [21,22]. While cermets perform well with pure H_2_ as a fuel, the presence of traces of SH_2_ or other impurities tends to deactivate them rapidly [23]. Alternative anode materials have been explored to prevent these poisoning problems [24]. An appealing solution is the use of monophasic oxide materials that combine the mixed ionic and electronic properties of cermets. For this reason, they are denominated mixed ionic and electronic conductor (MIEC) oxides [25,26]. Oxides with ABO_3_ perovskite structure are well known to exhibit the required properties when correctly choosing A and B metals [27,28]. In particular, derivatives from SrMoO_3_ have been shown to exhibit adequate properties and stability to work as anode materials in SOFC [29,30,31,32,33]. SrMoO_3_ itself is an excellent metallic band conductor [34], containing Mo^4+^ ions, which are stable under the reducing conditions created by the presence of the fuel at elevated temperatures. This ion is well-known to catalyze the oxidation reaction of the fuel, thus accomplishing its role as anode material. To enhance the ionic conductivity of this perovskite, which occurs by an oxygen vacancy mechanism, it is pertinent to increase the number of oxygen vacancies. This can be achieved by suitably doping the Mo sites with aliovalent elements, in materials of SrMo_1−x_B_x_O_3−δ_ composition [28,29,30,31,32].

In this paper, we describe the series of SrMo_1−x_Mn_x_O_3−δ_ oxides, with Mn being a suitable element to replace Mo. It is well known that Mn can adopt several charge states [35,36]. In this case, Mn is chosen because it has a suitable ionic size to adopt the octahedral position of a perovskite, and it can be an excellent replacement for Mo^4+^ in SrMoO_3_. The reducing conditions where this perovskite oxide is obtained (in forming gas at 1050 °C) are suitable to stabilize, nominally, the trivalent oxidation state of Mn. Perovskite materials with Mn^4+^ require the presence of air in the syntheses, whereas Mn^2+^ is too voluminous to comfortably coordinate in the octahedral sites of these oxides. For instance, rare-earth (R) manganites (RMnO_3_) are very well known and must be prepared under inert or reducing conditions [37]; otherwise, the preparation in air or O_2_ leads to RMnO_3+δ_ that indeed contain Mn^4+^, given the strong trend of Mn to oxidize just by thermal treatments in air [38].

For these SrMo_1−x_Mn_x_O_3−δ_ materials, we report on the synthesis of porous specimens by wet-chemistry techniques, their characterization involving X-ray diffraction and neutron diffraction, to evaluate the structural evolution upon doping, the identification of the transport properties relevant for their behavior as electrodes, as well as the magnetic behavior derived from magnetic Mn ions, and finally the performance in test cells supported on LSGM electrolyte and fed by H_2_ as a fuel.

## 2. Materials and Methods

### 2.1. Synthesis

The oxides of the family SrMo_1−x_Mn_x_O_3−δ_ (x = 0.05, 0.10, 0.15, 0.20) were synthetized via the citrate method. Sr(NO_3_)_2_, MnCO_3_, and (NH_4_)_6_Mo_7_O_24_·4H_2_O were weighed in the appropriate amounts and dissolved in a 10% citric acid solution. This mixture was heated at 300 °C and stirred at 375 rpm until a gel was formed. This gel was dried at 100 °C and calcined and decomposed in an oven at 600 °C for 12 h to form the scheelite precursors (SrMo_1−x_Mn_x_O_4−δ_). Subsequently, the obtained powder is reduced at 1050 °C with forming gas (5% H_2_/95% N_2_) in a tubular furnace for 13 h in a reducing environment to form the perovskite oxide SrMo_1−x_Mn_x_O_3−δ_ in its active phase.

### 2.2. Structural Characterization

Both scheelite and perovskite phases were characterized using a Bruker D8 Advance diffractometer (40 kV, 30 Ma, Bruker, Billerica, MA, USA), operated with DIFFRAC.SUITE software V7 in Bragg–Brentano reflection geometry. Cu Kα radiation (λ = 1.5416 Å) and a position-sensitive detector (PSD) were used, with a nickel filter to completely eliminate Cu Kβ radiation. XRD patterns were recorded over a 2θ range of 10 to 64°.

The thermal behavior of the crystallographic structure was investigated via NPD at temperatures of 25 °C, 100 °C, 200 °C, 300 °C, 400 °C, 500 °C, 600 °C, and 800 °C. NPD measurements were performed using the D1B diffractometer at the Institut Laue–Langevin in Grenoble, using a wavelength of λ = 1.280 Å and a 2θ range from 10 to 130°. Approximately 2 g of the sample was placed in a vanadium sample holder, and for the high-temperature measurements, it was placed in an oven working under vacuum. Each measurement took about 2.5 h. The diffractograms were analyzed via Rietveld refinement [39] using the FullProf suite software version 5.20. A pseudo-Voigt function was applied to model the diffraction peak shapes. Refinements included parameters such as scale factor, background points, zero shift, half-width, asymmetry-corrected pseudo-Voigt parameters, unit-cell dimensions, positional coordinates, and isotropic displacement factors for Sr, Mo, and Mn atoms, as well as anisotropic factors for all oxygen atoms. The coherent scattering lengths were 7.02, 6.715, −3.73, and 5.803 fm for Sr, Mo, Mn, and O, respectively.

### 2.3. Microstructural Characterization

For the single-cell evaluation, microstructural characterization was performed by capturing scanning electron microscopy (SEM) images using an FEINova NanoSEM 230 (FEI, Lausanne, Switzerland) field-effect microscope in addition to a Hitachi TM-1000 table-top microscope (Hitachi, Tokyo, Japan).

### 2.4. Thermogravimetric Analysis (TGA)

TGA was performed using a Mettler TA-3000 system (Barcelona, Spain) with a TC10 processor unit, and the thermogravimetric curves were collected in an O_2_ flow. The analysis began with the reduced perovskite phases, using around 40 mg of sample. The samples were heated at a rate of 10 °C/min from room temperature to 900 °C, with the thermogravimetric data recorded by a TG50 microbalance.

### 2.5. Thermal Expansion Coefficients

Dilatometry experiments were carried out to measure the thermal expansion coefficient (TEC) of both perovskite and scheelite phases. Sintered pellets of the perovskite phases, approximately 7 mm in diameter and 1.5 mm in thickness, were prepared by uniaxial pressing of the powders and annealed at 1050 °C for 12 h in a H₂/N₂ (5%/95%) flow to prevent oxidation. For the scheelite phases, pellets of similar size were sintered at 1050 °C for 12 h in air. The thermal expansion of these sintered samples was measured using a Linseis L75HX1000 dilatometer (Linseis, Selb, Germany) over a temperature range from 25 to 900 °C, with the perovskite samples analyzed under a forming gas (N_2_/H_2_ 95/5%) flow and the scheelite samples measured in an air atmosphere. The heating rate for all measurements was 10 °C min^−1^.

### 2.6. DC-Conductivity

Electrical conductivity was measured on sintered rectangular bar-shaped samples (approximately 2 × 3 × 9 mm) in the temperature range from 25 to 850 °C, using the four-point DC method with currents between 0.01 and 0.5 A. The pellets were prepared by pressing the powder in a Retsch PP25 Hydraulic Press (Haan, Germany) and sintering at 1050 °C for 12 h in a H_2_/N_2_ (5%/95%) atmosphere. Four platinum wires were attached to the samples in a four-point configuration using platinum paste, followed by calcination at 850 °C for 1 h under a H_2_/N_2_ flow. Electrical measurements were taken using a Potentiostat–Galvanostat AUTOLAB PGSTAT 302 from EcoChemie (Utrecht, The Netherlands), with data collected every 50 °C. For both dilatometry and conductivity measurements, the perovskite oxide pellets were sintered in a reducing atmosphere, while the scheelite oxide pellets were sintered in air.

### 2.7. Single-Cell Performance

The power density was evaluated in electrolyte-supported single cells consisting of an anode layer (SrMo_1−x_Mn_x_O_3−δ_) (SMMO), a La_0.4_Ce_0.6_O_2−δ_ (LDC) buffer layer, a La_0.8_Sr_0.2_Ga_0.83_Mg_0.17_O_3−δ_ (LSGM) electrolyte, and a SrCo_0.8_Fe_0.2_O_3−δ_ (SCFO) cathode. Test single cells were built with the configuration SMMO|LDC|LSGM|SCFO. LSGM was synthesized by heating La_2_O_3_, SrCO_3_, Ga_2_O_3_, and MgO powders at 1000 °C and 1200 °C for 20 h each, with intermediate grinding. The final LSGM pellets were obtained by pressing the powders into pellets with a uniaxial dam and sintering at 1450 °C for 20 h in air. After grinding, the pellets had a thickness of about 0.3 mm. Inks of LDC, SMMO, and SCFO were deposited on the electrolyte surface. LDC was first printed onto one side of the LSGM disk and fired at 1300 °C for 1 h. SMMO was then printed onto the LDC layer and fired at 1100 °C, followed by SCFO printing and sintering at 1100 °C for 1 h. The resulting electrodes had a thickness of 5 μm, and the effective electrode area was 0.25 cm^2^. Platinum gauze was applied to both the anode and cathode as current collectors. The single cell was tested in a vertical tubular furnace at 850 °C, with the anode exposed to a flow of H_2_ (20 mL min^−1^) and the cathode exposed to air. Electrochemical characterization was performed using an AUTOLAB 302N Potentiostat/Galvanostat (Utrecht, The Netherlands), with voltage scans from 1.3 V to 0.1 V, with 0.010 V steps, and holding 10 s at each step. Current density was calculated from the recorded current through the effective area of the cell (0.25 cm^2^). Each voltage-current scan was considered one cycle, with cell activation monitored over subsequent cycles until maximum power output was achieved.

### 2.8. Magnetic Measurements

The magnetic properties were analyzed using a SQUID magnetometer (MPMS-3) from Quantum Design (San Diego, CA, USA), across a temperature range of 1.8 to 370 K, with applied magnetic fields up to 7 T.

## 3. Result and Discussion

### 3.1. Crystallographic Characterization

**Figure 1:**
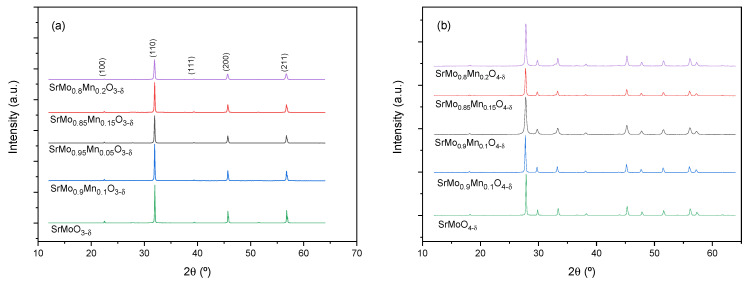
X-ray diffractograms of the perovskite (**a**) and scheelite (**b**) phases for the members of the SrMo_1−x_Mn_x_O_3−δ_ and SrMo_1−x_Mn_x_O_4−δ_ families of compounds, respectively.

Both the scheelite and perovskite phases are characterized by XRD. Figure 1 shows the diffractograms of the different samples of the SrMo_1−x_Mn_x_O_3−δ_ family and SrMo_1−x_Mn_x_O_4−δ_ as-obtained precursors. As can be seen, the phases obtained are very crystalline and pure.

### 3.2. Neutron Powder Diffraction (NPD)

**Figure 2:**
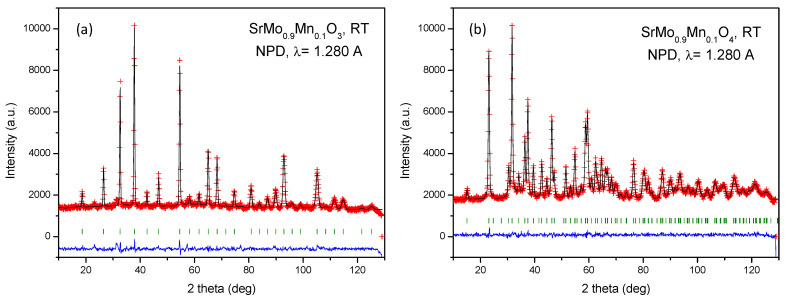
Observed (crosses), calculated (full line), and difference (at the bottom) NPD profiles for (**a**) reduced SrMo_0.9_Mn_0.1_O_3−δ_ perovskite and (**b**) oxidized SrMo_0.9_Mn_0.1_O_4−δ_ scheelite at 25 °C, refined in the cubic Pm-3m and tetragonal I4_1_/a space groups, respectively. The vertical markers indicate the allowed Bragg reflections.

**Figure 3:**
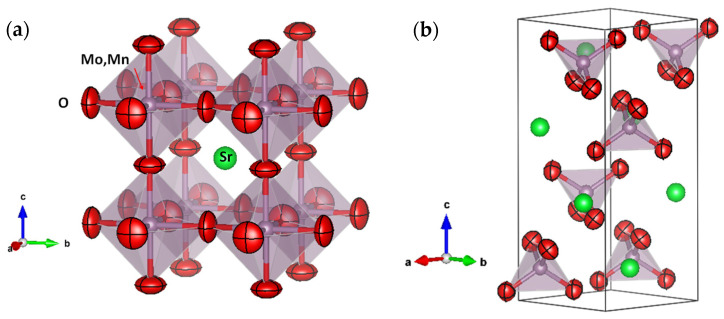
View of (**a**) cubic perovskite crystal structure, where the green sphere is the Sr atom, the red flattened spheres are the anisotropic (oblate) oxygen ellipsoids, and the grey spheres are the (Mo,Mn) atoms, and (**b**) precursor scheelite structure (oxidized specimen), with the same color code.

Neutrons are an effective probe to identify oxygen vacancies in oxides, due to the suitable scattering factor for O atoms in the perovskite structure. Moreover, NPD is a powerful tool to quantify the concentration of oxygen vacancies, which in combination with complementary optical analysis can define the exact nature of the vacancy. We performed an NPD study at room temperature (RT) of both perovskite (reduced) and scheelite (oxidized) phases for a selected member of the series with a manganese doping level x = 0.1. Figure 2a and Figure 2b show the good agreement between the observed and calculated NPD patterns at room temperature for the SrMo_0.9_Mn_0.1_O_3−δ_ perovskite and SrMo_0.9_Mn_0.1_O_4_ scheelite crystal structures, respectively. The cubic perovskite structure was refined by the Rietveld method in the *Pm*-3*m* space group (No. 221), Z = 1, with Sr atoms located at 1*b* (½, ½, ½) sites, Mo and Mn atoms randomly distributed at 1*a* (0, 0, 0) sites, and the O oxygen atoms located at 3*d* (½, 0, 0) positions. The determined unit-cell parameter is *a* = 3.9491(1) Å. This is smaller compared to the unit-cell magnitude of pristine SrMoO_3_, of 3.97629(3) Å [40], due to the smaller size of Mn^3+^ ions (0.58 Å) compared to Mo^4+^ (0.65 Å) in octahedral coordination. Remarkably, the contraction of the unit cell upon Mn doping excludes the possibility that the introduced ions are Mn^2+^, which are too large (0.83 Å) and point to the mentioned low-spin Mn^3+^ ions. The Mo/Mn and O occupancies were refined; interestingly, Mo and Mn scattering factors are strongly contrasting, and the determination yields a stoichiometry close to the nominal one. A full oxygen stoichiometry is observed at RT. The crystallographic formula is SrMo_0.907(5)_Mn_0.093(5)_O_3.003(1)_. The anisotropic displacement factors of O were also refined and are included in Table 1. They are also shown in Figure 3a, presenting flattened ellipsoids (oblate type) that correspond to the highly covalent (Mo,Mn)-O bonds.

It is remarkable that the oxygen stoichiometry of the perovskite phase is close to the expected value of 3.00, despite the introduction of a lower-valence element, such as Mn, in a trivalent state according to the structural and magnetic data, into the Mo^4+^ positions. The reason behind this finding is probably the valence variability of Mo, being able to adopt a mixed Mo^4+^ − Mo^5+^ valence to accommodate the introduction of Mn (hole doping effect). For instance, a mixed valence of Mo^4.10+^, implying 10%Mo^5+^ + 90%Mo^4+^ would correspond to the mentioned crystallographic stoichiometry SrMo_0.907(5)_Mn_0.093(5)_O_3.003(1)_ for the nominal x = 0.1 compound, at room temperature.

Regarding the crystal structure of the precursor scheelite phase, with nominal composition SrMo_0.9_Mn_0.1_O_4_, it was also refined from NPD data at RT. The structure is tetragonal, with *I4_1_/a* symmetry (No. 88, origin at −1). In this setting, Sr is located at 4*b* (0, 1/4, 5/8) sites, Mo is partially substituted by Mn, statistically distributed in the Wyckoff position 4*a* (0, 1/4, 1/8), and the oxygen is placed at 16*f* (x, y, z) positions. In the crystal, Sr is coordinated to 8 oxygen atoms (4 short and 4 long), whereas Mo and Mn are coordinated to 4 oxygen atoms in a regular tetrahedral arrangement. The quality of the Rietveld fit is displayed in Figure 2b, showing an excellent agreement between observed and calculated profiles. Supplementary Table S1 contains the atomic parameters after the Rietveld refinement of the scheelite phase for x = 0.1. Figure 3b shows a view of the scheelite structure, highlighting the anisotropic displacement factors of oxygen atoms.

### 3.3. High Temperature NPD

**Figure 4:**
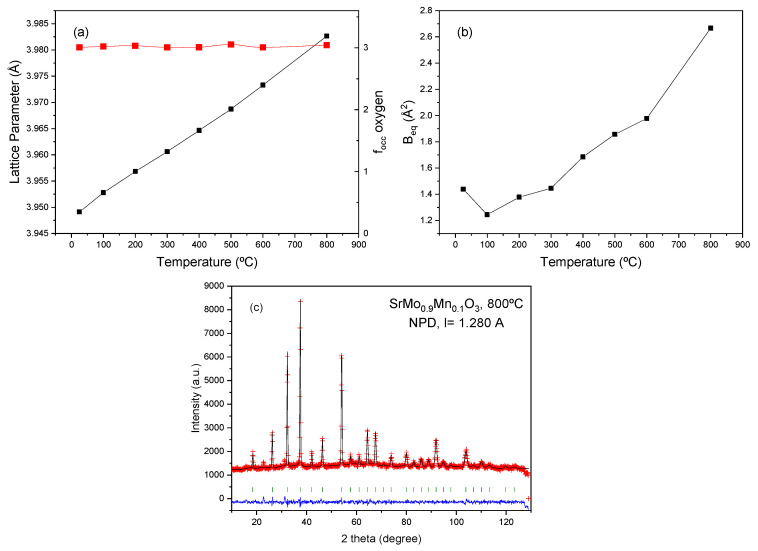
Evolution of different atomic parameters with temperature. The error bars are smaller than the size of the symbols. (**a**) Left axis: unit cell parameters; right axis: oxygen content of the perovskite unit cell; (**b**) equivalent isotropic displacement factor for O atoms; (**c**) Rietveld plot at 800 °C; the red crosses are the experimental points; the black continuous line is the calculated profile; the difference is at the bottom, and the green tick marls are the Bragg positions.

It is interesting to evaluate the thermal evolution of the crystal structure in order to assess the presence of oxygen vacancies at the working temperature of these anode materials within an SOFC. For this purpose, a temperature-dependent NPD study was performed on the x = 0.1 compound. Additional NPD patterns were collected at 100, 200, 300, 400, 500, 600, and 800 °C in a vanadium furnace operating under vacuum, coupled with the neutron diffractometer. The crystal structure remains cubic in the entire temperature interval, and it could be refined using the same crystallographic model, in the *Pm-3m* space group. Figure 4a displays the thermal evolution of the unit-cell parameter for the perovskite phase, which regularly increases with temperature, as expected. A thermal expansion coefficient (TEC) of 12.02 × 10^−6^ K^−1^ is estimated between 25 and 800 °C for the perovskite oxide. This is in reasonable agreement with the value determined from dilatometry measurements for the SrMoO_3_ family, as described below. Figure 4b shows the equivalent isotropic displacement (B_eq_) factors for O atoms that increase progressively with temperature, from 1.20 Å^2^ at 100 °C to 2.62 Å^2^ at 800 °C. Figure 4c illustrates the goodness of the fit at 800 °C, showing an excellent agreement between observed and calculated profiles. The occupancy factor of oxygen positions (right axis of Figure 4a) seems to be invariant with temperature, within the standard deviations. Even at 800 °C, no oxygen vacancies are determined, confirming the non-defective nature of the oxygen sublattice of this perovskite under the SOFC working conditions. This is an unexpected behavior, since the doping of the Mo sites of SrMoO_3_ with low valence elements such as Mn was intended to create a substantial number of oxygen vacancies, thus promoting the mobility of O^2-^ ions across the electrode. This result, determined by neutron diffraction, is invaluable to account for the properties of this family of materials as plausible electrodes in SOFCs. Although the trend of many transition metal oxides when heated in reducing conditions (or vacuum) is to release oxygen atoms by a progressive reduction of the mentioned metals, this trend is overcome in this case by internal disproportionation reactions. The variability of the valences of both Mn and Mo ions plays a role in this process, keeping the oxygen sublattice unchanged. In very similar examples, where SrMoO_3_ perovskite has been doped with fixed-valence elements (like Mg^2+^, Al^3+^, Ga^3+^…) [31,32], the neutron diffraction technique indeed showed a progressive increment of the oxygen vacancies, thus favoring the oxygen mobility and their MIEC behavior. This structural result accounts for the relatively poor performance of the SrMo_1−x_Mn_x_O_3−δ_ materials as anodes in SOFC.

### 3.4. Electrical Conductivity

**Figure 5:**
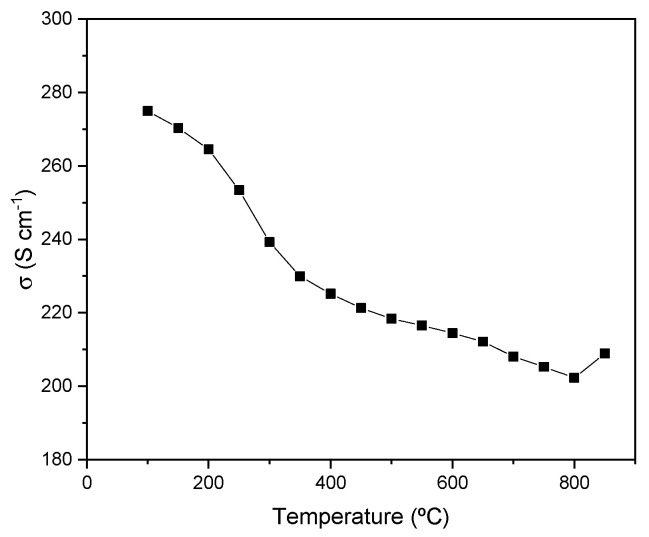
Electrical conductivity of SrMo_0.9_Mn_0.1_O_3−δ_ as a function of temperature measured in a forming gas flow (H_2_/N_2_ 5%/95%).

Figure 5 illustrates the temperature-dependent electrical conductivity of SrMo_0.9_Mn_0.1_O_3−δ_. Under reducing conditions, a metallic-like conductivity is observed across the entire temperature range measured. A value of 209 S/cm was determined at 850 °C, which surpasses other values obtained in the literature for anode materials with metallic-like behavior [31,32,41].

### 3.5. Thermal Expansion Behavior

**Figure 6:**
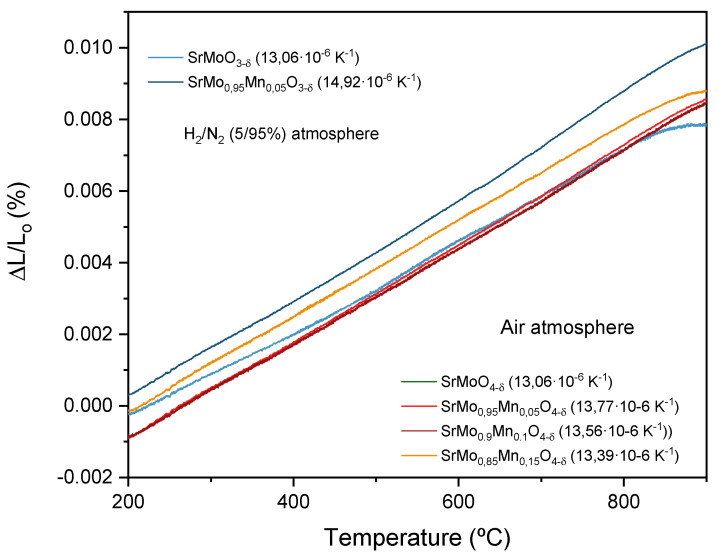
Thermal expansion measured by dilatometry for SrMo_0.95_Mn_0.05_O_3−δ_ perovskites, compared to the non-doped sample, and the SrMo_1−x_Mn_x_O_4−δ_ scheelite family compared to the non-doped sample.

To assess the mechanical compatibility of the components in the single cell, thermal expansion measurements were performed. The pellets, prepared as described in the experimental section, were subjected to temperature cycles ranging from 25 to 900 °C, and the changes in their thickness during heating were recorded. Using these data, the coefficient of thermal expansion (TEC) was determined for each sample. The results showed a steady, monotonic thermal expansion, with no sudden changes or discontinuities that could compromise the mechanical integrity of the cell. Values for TEC are shown in Figure 6. These values are similar to the TEC of the SrCo_0.8_Fe_0.2_O_3−δ_ cathode (13.4 10^−6^ K^−1^) [42], the La_0.4_Ce_0.6_O_2−δ_ buffer layer (12.0 10^−6^ K^−1^) [43], and the LGSM electrolyte (12.4 10^−6^ K^−1^) [44]. Therefore, we can anticipate good mechanical compatibility between the various components of the SOFC at the operating temperature.

### 3.6. Thermogravimetric Analysis

**Figure 7:**
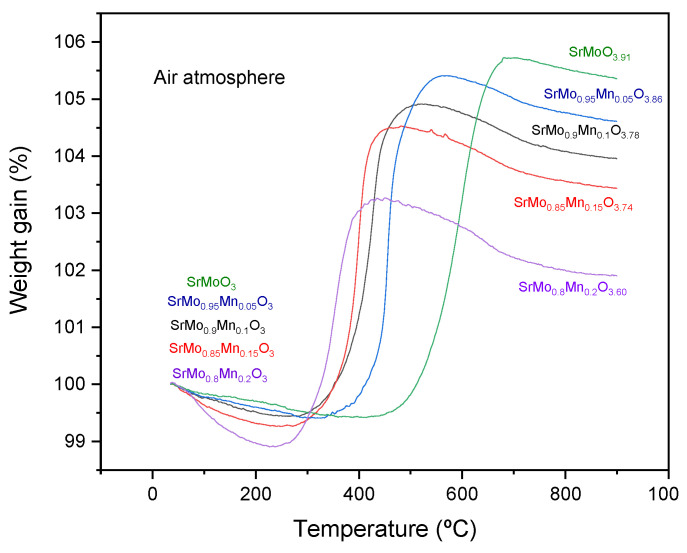
Thermogravimetric study for all SrMo_1−x_Mn_x_O_3−δ_ family compounds in air atmosphere.

The oxygen content variations of perovskites of the SrMo_1−x_Mn_x_O_3−δ_ were studied using TGA under an oxygen flow between 35 and 900 °C. Figure 7 shows a mass increase of between 6.3 and 4.3%, which translates into a gain of between 0.91 and 0.60 oxygen atoms per unit formula. As the proportion of dopant in the structure increases, the resulting scheelite phase has a lower proportion of oxygen, with x = 0.2 having the lowest number of oxygen atoms per unit formula. A similar behavior is obtained for all samples. From RT up to 300 °C, a slight decrease in weight is experienced, probably due to the desorption of oxygen on the surface of the powdered samples. Between 300 and 550 °C, a considerable increase in mass is observed, corresponding to the oxidation of the sample and its change from the reduced cubic phase to the oxidized tetragonal scheelite. After oxidation, the material stabilized up to 800 °C before showing a slight mass decrease. The oxidation and reduction cycles of the material were found to be fully reversible with minimal volume changes, as both the perovskite (with Mo^4+^ − Mo^5+^ oxidation states) and scheelite phases (with Mo^6+^ oxidation state) exhibited similar thermal expansion properties, preventing issues like cracking or delamination during cycling.

### 3.7. Single-Cell Power Tests

**Figure 8:**
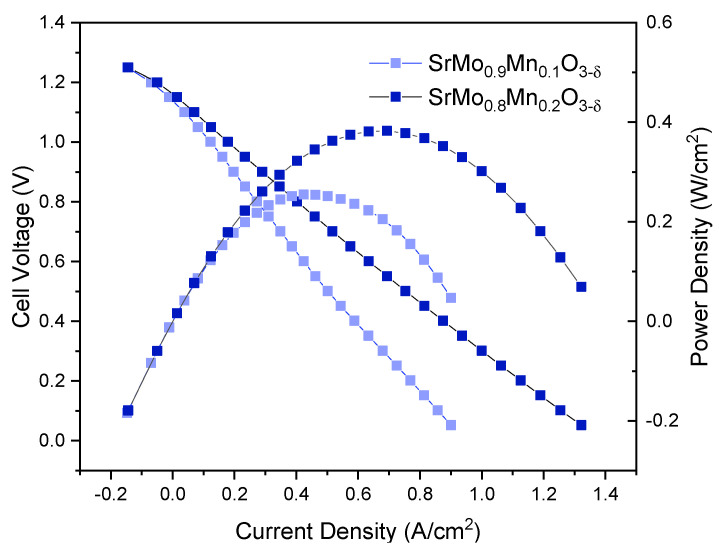
Cell voltage (on the left axis) and power density (on the right axis) as a function of current density for SrMo_0.9_Mn_0.1_O_3−δ_ and SrMo_0.8_Mn_0.2_O_3−δ_ anodes in a single cell featuring the anode/LDC/LSGM/SCFO configuration, measured in pure H_2_ at 850 °C.

An electrolyte-supported SOFC was assembled for each sample. LSGM electrolyte pellets, prepared using the ceramic method and sintered at 1450 °C, were polished to a thickness of approximately 300 μm. A buffer layer (LDC) and the corresponding anode were deposited on one side of the pellet, while a reference cathode (SCFO) was applied to the opposite side. Platinum grids were stuck to both sides using platinum ink as current collectors, with platinum wires attached to each grid and connected to an external circuit to measure power output. The results of these tests are shown in Figure 8. The product of the cell voltage and current density yielded a maximum power density of 255 mW/cm^2^ for x = 0.1 and 382 mW/cm^2^ for x = 0.2, as depicted on the right axis of Figure 8.

### 3.8. Chemical Compatibility

**Figure 9:**
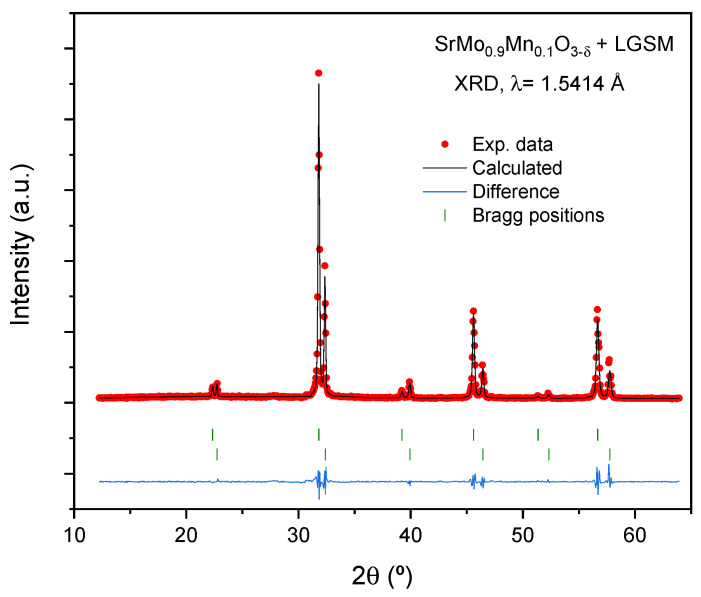
Rietveld-refined X-ray diffraction (XRD) diffractograms obtained for mixtures of SrMo_0.9_Mn_0.1_O_3−δ_ and LSGM after thermal treatment at 1050 °C in a H_2_(5%)/N_2_ atmosphere for 10 h. The analysis revealed no reaction products between the phases, since the diffraction peaks correspond solely to the original reactants. The diffraction patterns showed two distinct sets of Bragg peaks corresponding to the anode (SrMo_0.9_Mn_0.1_O_3−δ_) and LSGM phases, with no evidence of any novel phase. The unit-cell parameters of both phases remained unchanged.

Equimolar amounts of electrolyte and anode were mixed and heated at 1050 °C for 10 h in forming gas. The result is shown in the diffraction pattern of Figure 9. As shown, no additional peaks are observed, other than those from the initial compounds, both of which are cubic perovskites. This implies that, after the thermal treatment, there are no symptoms of a chemical reaction between the initial phases.

### 3.9. Magnetic Measurements

**Figure 10:**
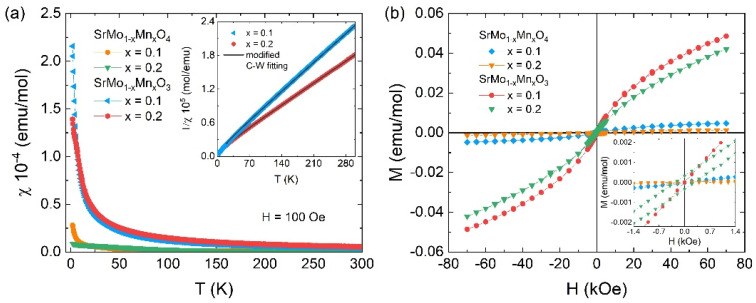
(**a**) Temperature-dependence magnetic susceptibility *χ*(*T*) at FC protocol for SrMo_1−x_Mn_x_O_4_ and SrMo_1−x_Mn_x_O_3_ (x = 0.1 and 0.2) under an external magnetic field of 100 Oe. The insert shows the *χ*^−1^(*T*) curves zoom and their fitting (black line) by the modified Curie–Weiss law χ(T) = χ_0_ + C/(T − Θ) for SrMo_1−x_Mn_x_O_3_ (x = 0.1 and 0.2). (**b**) Isotherm *M*(*H*) curves recorded at 1.8 K for both samples, respectively.

For the study of the magnetic properties of both oxidized and reduced oxides, we recorded the temperature-dependent magnetic susceptibility χ(T) and isotherm magnetization M(H) curves. Figure 10a exhibits the χ(T) curves from 1.8 up to 300 K at the FC protocol for SrMo_1−x_Mn_x_O_4_ and SrMo_1−x_Mn_x_O_3_ (x = 0.1 and 0.2) under an external magnetic field of 100 Oe. For the oxidized SrMo_1−x_Mn_x_O_4_ samples, the FC susceptibility curves behave almost constantly with temperature, presenting a χ ≈ 0.6 × 10^−6^ emu/mol value at room temperature, which slowly increases to lower temperatures to a maximum of ~0.28 × 10^−4^ emu/mol at 1.8 K (for x = 0.1), matching the onset of short-range spin–spin correlations between Mn–Mn interactions distributed at random.

For the reduced SrMo_1−x_Mn_x_O_3_ samples, the *χ*(*T*) curves maintain a similar paramagnetism-like behavior. However, a significant magnitude increase is observed mainly at lower temperatures, achieving a magnetic susceptibility value of ~2.2×10^−4^ emu/mol (x = 0.1) at 1.8 K (Figure 10a). In this case, we fitted the inverse of susceptibility χ^−1^(T) curves in the 20–300 K range for SrMo_1−x_Mn_x_O_3_ (x = 0.1 and 0.2) according to the modified Curie–Weiss (C-W) law, χ(T) = χ_0_ + C/(T − Θ), where χ_0_ is temperature-independent susceptibility, C is the Curie constant, and Θ is the Weiss temperature [45], as shown in inset of Figure 10a. The fitting yields χ_0_ ≈ 9.3 × 10^−4^ and 7.5 × 10^−4^ emu/mol as well as Θ ≈ −19 and −25 K for x = 0.1 and 0.2, respectively. The obtained χ_0_ values are consistent with those reported for other similar materials [45,46]. The negative Weiss temperature indicates a predominance of antiferromagnetic (AFM) interactions for both samples.

According to Zhang et al. [46], the electrons of Mo ions in SrMo_1−x_Mn_x_O_3_ are itinerant, and only Mn^3+^ magnetic ions are expected to be localized within the sample. We obtain effective paramagnetic moments of μ_eff_ ≈ 0.26 μ_B_ (x = 0.1) and 0.29 μ_B_ (x = 0.2) from μ_eff_ = $\sqrt{8C}$, which are smaller than those theoretically expected. We hypothesize that the reduced samples present Mn^3+^ in a low spin state with S =1 [47], resulting in μ_theo_ ≈ 0.89 and 1.27 μ_B_ for x = 0.1 and 0.2, respectively (from μ_theo_ $=\sqrt{x{\left(\mu_{Mn^{3+}}\right)}^{2}}$), which are larger than the μ_eff_ values. In this case, the Mn doping in SrMoO_3_, where Mo^4+^ electrons are delocalized (and are responsible for the χ_0_ temperature-independent susceptibility term), induces a weakening of the local Mn^3+^ magnetic moments by partial electron delocalization into the Mo-4*d* orbitals. Consequently, the possible presence of competition between AFM and ferromagnetic (FM) interactions of these local moments leads to smaller μ_eff_ compared with μ_theo_. This feature can be visualized through the M(H) curves exhibited in Figure 10b, where the non-saturated hysteresis behavior at low temperatures (1.8 K) suggests the coexistence of AFM and FM-like short-range interactions. Furthermore, the increased magnetization and small hysteresis (inset of Figure 10b) observed for the reduced samples, which is similar to other molybdates such as SrMo_1−x_R_x_O_3_ (R = Cr, Ni, Fe) [45,48,49], corroborate with enhanced magnetic susceptibility at low temperatures by the combination of the Mo reduction and Mn-doping in SrMoO_4_.

### 3.10. SEM Post Mortem

**Figure 11:**
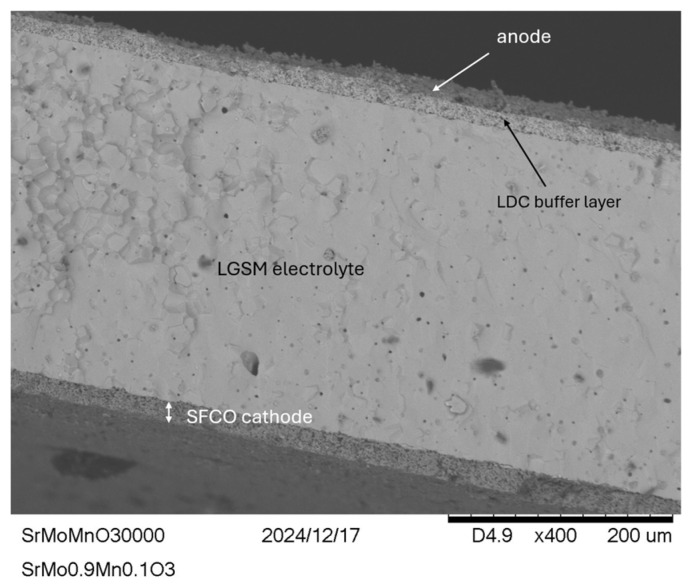
SEM images of the SrMo_0.9_Mn_0.1_O_3−δ_ test cell after testing, where the anode and cathode layers are well-defined and securely attached. There is a noticeable porosity, which is crucial for the fuel cell function. On both sides of the electrolyte, the cathode layer as well as the anode and the buffer layer can be distinguished.

The tested single cells were examined by SEM. Figure 11 shows a cross-sectional image of one of them. As shown, no cracks or delaminations are visible in any of the components, which is consistent with previous tests. Additionally, the different layers of the single cell are clearly defined and do not mix with each other. Both the anode, reference cathode, and buffer layer exhibit a certain porosity, which is a fundamental requirement for the operation of the fuel cell.

## 4. Conclusions

### Neutron Powder Diffraction (NPD)

In this study, a series of perovskite-based materials with nominal SrMo_1−x_Mn_x_O_3−δ_ stoichiometry were successfully synthesized and evaluated as anode materials for SOFCs. These oxides, obtained via sol-gel synthesis, demonstrated poor performance in SOFC tests, with power densities reaching 232 and 382 mW/cm^2^ at 850 °C for the x = 0.1 and 0.2 Mn doping levels, respectively. This behavior was attributed to the lack of oxygen vacancies generated by Mn doping, as demonstrated in a temperature-dependent neutron diffraction study. The valence variability of Mn and Mo ions suggests internal disproportionation processes, resulting in a full oxygen sublattice O_3_ of the perovskite specimens at temperatures as high as 800 °C.

Furthermore, the magnetic measurements indicate strong electron delocalization, thus weakening the magnetic moments associated with Mn^3+^ ions, which do not behave as isolated moments in a diamagnetic matrix. Although the thermal expansion coefficients, transport properties, chemical compatibility, and post-mortem studies are suitable, the structural results suggest that Mn-doped SrMoO_3_ perovskites cannot be considered as MIEC oxides, as they lack adequate ionic conductivity. Despite the observed limitations, the findings of this study underscore the importance of developing strategies that improve oxygen vacancies and ionic conductivity, which could lead to significant advancements in SOFC technology. Future research could explore alternative doping strategies or composite materials that may address these issues, driving the development of more efficient anode materials for SOFCs.

## Figures and Tables

**Table 1:** Unit-cell, atomic coordinates, and displacement parameters for SrMo_0.9_Mn_0.1_O_3−δ_ refined in the cubic Pm-3m (no. 221) space group from NPD at RT. * corresponds to U isotropic; otherwise it is U equivalent.

	x	y	z	U_iso_ */U_eq_ (Å^2^)	Occ. (<1)
Sr	0.50000	0.50000	0.50000	0.0129 (10) *	
Mo	0.00000	0.00000	0.00000	0.0075 (11) *	0.907 (5)
Mn	0.00000	0.00000	0.00000	0.0075 (11) *	0.093 (5)
O	0.50000	0.00000	0.00000	0.0180 (13)	1.003 (6)
Atomic displacement parameters (Å^2^)					
	U11	U22	U33	U12	U13
O	0.0077 (15)	0.0232 (11)	0.0232 (11)	0.00000	0.00000

## Data Availability

The original contributions presented in the study are included in the article, further inquiries can be directed to the corresponding author.

## Supplementary Materials

The following supporting information can be downloaded at: https://www.mdpi.com/article/10.3390/ma18030542/s1, Table S1: structural parameters of the scheelite SrMo_0.9_Mn_0.1_O_4_ structure at RT, from NPD data.
