# Supplementary material for: Valence Variability Induced in SrMoO₃ Perovskite by Mn Doping: Evaluation of a New Family of Anodes for Solid-Oxide Fuel Cells
Source: Materials (Basel). 2025 Jan 24;18(3):542. doi: 10.3390/ma18030542 (PMC11818537; doi:10.3390/ma18030542)
Supplement: Supplementary file 1 [file materials-18-00542-s001.zip › materials-3412661-supplementary.pdf]

SUPPLEMENTARY INFORMATION

Valence Variability Induced in $SrMoO_3$ Perovskite by Mn Doping: Evaluation of a New Family of Anodes for Solid-Oxide Fuel Cells

Lucía Sánchez de Bustamante 1, Romualdo Santos Silva, Jr. 2, José Luis Martínez 1, María Teresa Fernández-Díaz 3, Ainara Aguadero 1 and José Antonio Alonso 1,*

[1] Instituto de Ciencia de Materiales de Madrid, Consejo Superior de Investigaciones Científicas, Cantoblanco, E-28049 Madrid, Spain; lucia.sb@csic.es (L.S.d.B.); martinez@icmm.csic.es (J.L.M.); ainara.aguadero@csic.es (A.A.)

[2] Departamento de Física de Materiales e Instituto Pluridisciplinar, Universidad Complutense de Madrid, E-28040 Madrid, Spain; romualdo.silva@csic.es

[3] Institut Laue Langevin, BP 156X, F-38042 Grenoble, France; ferndiaz@ill.eu

* Correspondence: ja.alonso@icmm.csic.es

Table S1. Unit-cell, atomic coordinates and displacement parameters for $SrMo_{0.9}Mn_{0.1}O_{3-\delta}$ refined in the tertagonal I41/a (No. 88, origin at -1) space group from NPD at RT, λ= 1.280 Å. a = 5.3545 (5) Å, c = 11.9503 (13) Å, V = 342.62 (6) Å3, Z = 4

Fractional atomic coordinates and isotropic or equivalent isotropic displacement parameters (Å^2)

	x	*y*	*z*	U_{iso}*/U_{eq}	Occ. (<1)
Sr	0.00000	0.25000	0.62500	0.01437 (11)*	
Mo	0.00000	0.25000	0.12500	0.00865 (9)*	0.9880 (9)
Mn	0.00000	0.25000	0.12500	0.00865 (9)*	0.0120 (9)
O	0.23827 (4)	0.11477 (4)	0.044660 (19)	0.0142 (11)	0.988 (10)

Atomic displacement parameters (Å^2)

	U^{11}	U^{22}	U^{33}	U^{12}	U^{13}	U^{23}
O	0.0138 (11)	0.0126 (13)	0.0162 (10)	0.0019 (12)	0.0021 (8)	0.0017 (10)
